# Genetically Guided Mediterranean Diet for the Personalized Nutritional Management of Type 2 Diabetes Mellitus

**DOI:** 10.3390/nu13020355

**Published:** 2021-01-25

**Authors:** Kalliopi Gkouskou, Evgenia Lazou, Efstathios Skoufas, Aristides G. Eliopoulos

**Affiliations:** 1 Department of Biology, School of Medicine, National and Kapodistrian University of Athens, Mikras Asias 75, 11527 Athens, Greece; evgelazou@gmail.com (E.L.); stathisskoufas@yahoo.gr (E.S.); 2 Embiodiagnostics Biology Research Company, 71305 Heraklion, Greece; 3 Center for New Biotechnologies and Precision Medicine, School of Medicine, National and Kapodistrian University of Athens, 15772 Athens, Greece; 4 Center of Basic Research, Biomedical Research Foundation of the Academy of Athens, 11527 Athens, Greece

**Keywords:** type 2 diabetes mellitus, Mediterranean diet, SNP, nutrigenetics, personalized nutrition, glycemia

## Abstract

The current consensus for the prevention and management of type 2 diabetes mellitus (T2DM) is that high-quality diets and adherence to a healthy lifestyle provide significant health benefits. Remarkably, however, there is little agreement on the proportions of macronutrients in the diet that should be recommended to people suffering from pre-diabetes or T2DM. We herein discuss emerging evidence that underscores the importance of gene-diet interactions in the improvement of glycemic biomarkers in T2DM. We propose that we can achieve better glycemic control in T2DM patients by coupling Mediterranean diets to genetic information as a predictor for optimal diet macronutrient composition in a personalized manner. We provide evidence to support this concept by presenting a case study of a T2DM patient who achieved rapid glycemic control when adhered to a personalized, genetically-guided Mediterranean Diet.

## 1. Introduction

Long considered a disease of minor significance, type 2 diabetes mellitus (T2DM) currently represents a major threat to human health with an estimated number of 425 million adult patients and four million deaths globally in 2017. Substantial evidence indicates that T2DM can be largely prevented or managed through adherence to a healthy lifestyle and a high-quality diet (as assessed by Healthy Eating Index-HEI [1]) is recommended to people at risk for diabetes or to T2DM patients as a therapy [2,3,4,5]. Surprisingly, however, there are no specific recommendations for the proportions of macronutrient intake that should be applied for people suffering from pre-diabetes or diabetes [6]. The Obesity Society, for example, recommends a diet comprising approximately 30% energy from total fat [15–20% from monounsaturated fatty acids (MUFA), 10% from polyunsaturated fatty acid (PUFA), 7% from saturated fatty acids (SFA)], 15–35% from protein and 45–65% energy from carbohydrates for the management of T2DM [7]. On the other hand, the Mediterranean diet (Med Diet) which demonstrates benefits over several metabolic diseases including T2DM, is considered a high fat diet (40% total fat) but rich in MUFA and poor in saturated fat [8,9,10].

Notably, the traditional Med Diet is rich in fibers and high-quality foods that have been proven a key strategy to achieve glycaemic control [11]. For example, grains, whole grain flours and breads from traditional Greek wheat varieties of *Triticum monococcum* and *Triticum dicoccum*, contain very high levels of alkylresorcinols [12] which have been associated with increased insulin sensitivity in metabolic syndrome patients [13] but are absent from white flour and its products.

It is therefore evident that there are no “golden rules” guiding the proportion of macronutrients for T2DM dietary therapy [14]. Moreover, even though the Med Diet has been proven successful in reducing chronic disease burden at population level [15], it does not take into account individualized needs and, as many other diets, it is based on population average. As a result, the search for an ideal high-quality diet for diabetes patients continues to grow.

In addition, T2DM is a highly heterogeneous disease influenced by genetic factors and nutrient-gene interactions, as indicated by twin and family studies [16,17,18]. With the emergence of genome wide association studies (GWAS) and more recently exome sequencing, a plethora of data has provided additional evidence for a genetic basis for T2DM. More than 140 genomic loci have been associated with predisposition to T2DM, improving our understanding of the genetic architecture and biology of the disease [19]. Genetics also influences individual responses to both macro- and micro-nutrients [20,21] and emerging evidence underscores the importance of gene–diet interactions in the improvement of glycaemic biomarkers.

We herein review the published evidence linking genetic variation to the selection of specific macronutrient composition for the management of T2DM (Table 1). Our overarching concept is to bridge the Med Diet with genetic information that can be used as predictor for optimal diet macronutrient composition in a personalized manner. We propose that by taking into account genetic information when formulating Med Diet-based nutritional recommendations, we can achieve better glycaemic control in T2DM patients. Herein we provide evidence to support this concept by presenting a case study of a T2DM patient who achieved rapid glycaemic control when adhered to a personalized, genetically-guided Med Diet.

## 2. Genetic Variations Guiding Carbohydrate Intake in T2DM

### 2.1. Genetic Variants Guiding the Quantity of Carbohydrate Intake

Even though carbohydrates (CHO) have a major dietary influence on postprandial blood glucose, evidence on total carbohydrate needs and T2DM is largely conflicting [26]. Surprisingly, even the anticipated protective effect of total fruit intake on T2DM could not be replicated in a meta-analysis contacted in the context of the European Prospective Investigation into Cancer (EPIC)-InterAct study [47]. Genetic variation is likely to account for the heterogeneity of T2DM patient responses to carbohydrates (Table 1). Indeed, several polymorphisms have recently been identified to influence glucose levels in response to carbohydrate intake. Among them, transcription factor 7-like 2 (*TCF7L2*) gene variant rs7903146 has attracted significant attention as it is one of the strongest genetic markers of predisposition to diabetes, with the TT genotype increasing disease risk by 40–50% [48]. Interestingly, even though this high risk polymorphism is associated with lower BMI ([49] and our unpublished data on Greek population), it is also associated with significantly higher increase of fasting glucose when consuming increased amounts of desserts compared to non-carriers [46].

The melatonin receptor 1b (*MTNR1B*) variant rs1387153 is another example of genetic variation influencing T2DM patient responses to CHO. An analysis of 5 cohort studies including more than 28,000 participants has demonstrated that the minor T allele of rs1387153 strongly interacts with CHO in modulating fasting glucose, to the extent that every 1% increase in CHO intake exacerbates the fasting glucose-raising effect of the T allele [22].

Cryptochrome 1 (*CRY1*) variant rs2287161, which has been associated with the regulation of circadian rhythms and linked to depression and sleep disturbance [50], also modifies the CHO effect on glycaemic indices. In this case, increased carbohydrate intake (as a percentage of energy intake) was associated with elevated HOMA-IR and fasting insulin only among individuals homozygous for the risk allele C [24]. In contrast, G homozygotes for pro-protein convertase subtilisin/kexin type 7 (*PCSK7*) gene variant rs236918 who were assigned to a high-carbohydrate diet displayed a greater decrease in fasting insulin levels and HOMA-IR than non-carriers [25]. Interestingly, both *MTNR1B* and *PCSK7* polymorphisms have been associated with increased risk of diabetes [51,52]. 

### 2.2. Genetic Variants Guiding the Quality of Carbohydrate Intake

The quality of carbohydrates appears to play important roles in the management of T2DM and the results of relevant prospective studies and clinical trials are much more coherent compared to those addressing the impact of the quantity of carbohydrates on T2DM [53]. People with diabetes and those at risk for diabetes are encouraged to consume at least the amount and quality of dietary fibre recommended for the general public that includes vegetables, pulses and fruit [54].

However, emerging evidence suggests that genetic variation may influence individual responses to different sources of fibre. Among them, *TCF7L2* gene variant rs7903146 has attracted significant attention [48]. Interestingly, two independent studies have reported that whole grain as a dietary source of CHO is associated with protection from T2DM only among non-risk CC genotype carriers [26,55]. This suggests that carriers of a T allele of rs7903146 would not benefit from a whole grain-enriched diet and alternative dietary approaches should be explored.

Likewise, the glucokinase regulatory protein (*GCKR*) gene variant rs780094, which has been explored as a component of polygenic risk for T2DM and dyslipidemia [56] diminishes the beneficial effects of whole-grain foods on insulin homeostasis, possibly via the reported effect of *GCKR* variant on both triglyceride and glucose levels [27].

Another gene polymorphism associated with the response of T2DM patients to particular sources of carbohydrates is apolipoprotein-A5 (*APOA5*) SNP rs964184. It has been reported that compared to rs964184 TT homozygotes, T2DM patients with impaired fasting glucose who carry the C allele of rs964184 could be more susceptible to the adverse effects of a high carbohydrate diet based on refined rice by having an elevation of triglycerides [23]. Therefore, the replacement of refined rice with whole grains and legumes in a high carbohydrate diet should be considered for individuals with impaired fasting glycemia who carry the *APOA5* rs964184 C variant, to prevent diabetic hypertriglyceridemia.

## 3. Genetic Variations Guiding Fat Intake in T2DM

Even though fat consumption has broadly been associated with elevated risk for the development of metabolic diseases, there is still a debate about the recommended daily intake of fat in T2DM patients, reportedly ranging from 10% up to 40% [14]. Conflicting data may be due to variations in food sources of fat [57] or the proportion of fat in the context of the overall dietary pattern. Indeed, according to the PREDIMED clinical study, individuals who adhered to a Med Diet plan supplemented with extra virgin olive oil had on average 40% lower risk to develop diabetes compared to those following a low-fat diet [58].

Several genes have been identified to impact responses to fat in T2DM. One of them is *PPM1K* (phosphatase, Mg^2+^/Mn^2+^-dependent 1 K) gene variant rs1440581. Individuals who are homozygous for the T allele of this SNP benefit most by a high-fat diet (40–45% fat, 15% protein, and 40–45% carbohydrate) in reducing insulin and homeostatic model assessment for β-cell function (HOMA-B) during weight loss whereas the opposite effect is observed for a low fat diet (20–25% fat, 15% protein, and 60–65% carbohydrate) [29]. Similarly, in another intervention study, the C allele was related to smaller decreases in serum insulin and homeostatic model assessment for insulin resistance (HOMA-IR) in obese and overweight individuals following an energy-restricted high-fat diet plan (40% energy intake from fat), whereas an opposite genotype effect on changes in insulin and HOMA-IR was observed in a low-fat diet group (20% energy intake from fat [28]). Together, these reports suggest that pro-diabetes or T2DM patients with *PPM1K* rs1440581 TT genotype would benefit from an energy-restricted high-fat diet whereas carriers of the C allele could be advised to follow a low-fat diet.

Genetic risk scores (GRS), generated by combining the additive effects of several genetic variants, have also been explored to predict optimal dietary fat composition for T2DM patients. For example, a high genetic risk score (GRS) of 14 fasting glucose-associated SNPs could predict health benefit for a diet low in fat [32]. Furthermore, in another study [30] a significant interaction between a genetic score calculated on the basis of eight variants previously associated with habitual coffee consumption [59] and dietary fat intake was observed. It was shown that the group with the highest genetic score for coffee consumption had significantly greater reduction in fasting insulin when following a low fat diet. Interestingly, even though the initial idea of this study was based on the fact that coffee consumption is associated with improved insulin sensitivity and reduced risk for diabetes [60], actual coffee consumption was not considered [30].

Variations in food sources of fat may play an important role in modulating fasting glucose and other bioclinical variables in T2DM patients. In general, replacing saturated with unsaturated fat in the diet reduces total cholesterol, LDL-C and CVD risk [61]. Surprisingly, a meta-analysis of observational studies has not identified high dietary saturated fatty acid (SFA) intake as a risk factor for T2DM [62] However, this conclusion should be considered through the prism of genetic variation. Indeed, high dietary SFA intake (≥15.5% energy) impairs insulin sensitivity and increases the risk for metabolic syndrome in carriers of the T risk allele of *TCF7L2* rs7903146 polymorphism relative to CC homozygotes [36] Likewise, high dietary SFA intake (≥11.8% energy) leads to increased waist circumference and consequently increases the risk for metabolic syndrome in carriers of the C risk allele of *CLOCK* rs1801260 [31]. Furthermore, diets rich in MUFA and PUFA may have a dual role as they can either reveal the protective effect that some polymorphisms have towards T2DM [35] or to abolish the detrimental effects of some others [34]. 

## 4. Genetic Variations Guiding Protein Intake in T2DM

Typically, the average daily level of protein intake for people with diabetes is 1–1.5 g/kg body weight/day or 15–20% of total calories. However, two published meta-analyses have indicated that high protein diets confer several health benefits in diabetes patients [63,64].

We reason that genetic variation may affect individual responses to different protein percentages in the diet (Table 1). Several studies support this notion. For example, polygenic scores, based on combinations of multiple susceptibility loci identified by GWAS, are increasing explored because they reflect the polygenic nature of T2DM and are more relevant to gene–diet interactions. A recent study demonstrated interaction between a polygenic score of 31 T2DM risk alleles and protein percentage in the diet. In particular, individuals with a lower polygenic score for T2DM showed more favourable responses to low-protein diets, including greater decreases in fasting insulin, HbA1c, and homeostatic model assessment for insulin resistance (HOMA-IR), and a lesser increase in and homeostatic model assessment for β-cell (HOMA-B) function, than did individuals with a higher risk score within 2 years of follow-up [38]. Another study similarly explored 159 obesity genes combined with phenotypic characteristics such as waist hip ratio and BMI to create different adiposity subtypes which were found to differentially modify the effect of protein intake on improving glucose metabolism [39].

An interaction between vitamin D metabolism-related variants and macronutrient responses in the context of T2DM was demonstrated by the Pounds Lost Trial group; patients with the vitamin D-increasing T allele of the *DHCR7* variant rs12785878 had a greater improvement of glycaemic parameters when assigned to high-protein diets, whereas improvement was not evident in those who followed a low protein diet [40].

## 5. Genetic Variations and Mixed Dietary Patterns for the Management of T2DM

A variety of dietary patterns are acceptable for the management of diabetes. Still, the evidence surrounding comparative benefits of different macronutrient ratios or dietary patterns in T2DM patients and the influence of genetics thereof is scarce [14]. 

One example of genetic variation influencing responses to mixed dietary patterns is *GIPR* variant rs2287019 (Table 1). T2DM patients who carry the T allele of rs2287019 and choose a low-fat, high-carbohydrate and high-fibre diet, show greater improvement in glucose homeostasis [42]. Likewise, it has been reported that T2DM patients carrying the *IRS1* variant rs2943641 CC genotype might obtain more benefits in weight loss and improvement of insulin resistance than those without this genotype when they adhere to a high-carbohydrate and low-fat diet [43]. Additionally, T2DM patients who are homozygous for the C allele of *S100A9* (S100 calcium-binding protein A9) rs3014866 variant are more likely to benefit from a low SFA: carbohydrate ratio intake to improve insulin sensitivity, based on HOMA-IR levels [41]. A western type diet, characterised by high intake of processed meat, red meat and heme iron, increases the risk of T2DM in individuals with a high polygenic score defined by ten T2DM risk alleles [45].

## 6. Discussion

Rapidly evolving technologies have offered unparalleled opportunities to assess bioclinical, dietary and genetic data that can improve the management of metabolic diseases such as T2DM. As indicated in this review, high-quality diets rich in *n*-3 PUFA and MUFA and low in saturated fat are universally proposed for the prevention and management of T2DM. This concept aligns with the Mediterranean dietary patterns typified by high-quality nutritional elements offering several health benefits. Notably, there is significant variation in the macronutrient distribution among different Mediterranean diets (Cretan/Greek, Italian, Spanish or Mediterranean-like) [65]. Likewise, dietary recommendations for T2DM patients largely vary with respect to macronutrient distribution which may impair the efficacy and goals of nutritional therapy for diabetes [14].

We propose that prevention and management of T2DM could be improved by combining genetically-guided quantitative and qualitative macronutrient recommendations (Table 1) with the high quality nutrition patterns of Med Diets. We have successfully applied this concept to several T2DM patients. As an example, a normal weight male T2DM patient, homozygous for *MTNR1B* rs1387153 risk allele T, with a high genetic risk score for diabetes and a high genetic score for coffee consumption was advised to change the macronutrient distribution of his diet from 54% carbohydrate, 32% fat and 16% protein to lower carbohydrate (50%), low fat (25%) and high protein (25%) diet based on Mediterranean high quality foods and olive oil (rich in MUFA and low in saturated fat) [66,67,68]. Some life style changes were also recommended based on his genetic makeup, such as not to consume dinner late at night and breakfast early in the morning and to avoid early rising (this individual was homozygous to *MTNR1B* rs10830963 risk allele G which is in linkage disequilibrium with rs1387153 in Greek people) [69,70,71]. Two months later he had significantly lower plasma insulin concentrations and higher insulin sensitivity.

The emerging prospects of precision nutrition call for re-evaluation of dietary therapies for T2DM taking into account genetic information that could enable stratification of patients towards specific macronutrient intakes. Intervention and observational studies should involve collection of extensive dietary exposure data and, importantly, validation in independent populations to provide solid evidence for interactions between genotype, diet and disease [72].

## Figures and Tables

**Table 1:** Gene variants associated with the selection of specific macronutrients for the dietary management of T2DM.

Gene	Variants	Macronutrient/s Involved in Health Outcome	Health Outcome Related to T2D	Cohort/Time	Reference
**Carbohydrates (CHO)**					
*MTNR1B* Melatonin receptor 1B	rs1387153 (C/T) T risk allele for T2D; C is the common allele	Increment of 1% of CHO	0.003 mmol/L higher fasting glucose with each additional 1% carbohydrate intake in the presence of the *MTNR1B* rs1387153 risk T allele	5 cohort studies including up to 28,190 participants of European descent from the Cohorts for Heart and Aging Research in Genomic Epidemiology (CHARGE) Consortium.	[22]
*APOA5* Apolipoprotein A5	rs662799 (T/C) C risk allele dyslipidemias; T is the common allele	Substitution of high-quality CHO with low quality (whole grains and legumes substitution with refined rice)	Patients with impaired glucose carrying the *APOA5* rs662799 risk allele C showed a greater increase in the mean percent changes of triglyceride and apolipoprotein A5 when they substituted whole grains and legumes with refined rice	93 patients with impaired glucose with 50 risk allele carriers/12 weeks	[23]
*CRY1* (Cryptochrome Circadian Regulator 1)	rs2287161 (G/C minus) C risk allele for mood disorders; G is the common allele	Increase in CHO intake (% of energy intake)	An increase in carbohydrate intake (% of energy intake) was associated with a significant increase in homoeostatic model assessment (HOMA-IR) for insulin resistance fasting insulin and a decrease in QUICKI only among individuals homozygous for the *CRY1* rs2287161 risk allele C	Two independent populations: a Mediterranean (*n* = 728) and a European origin North American population (*n* = 820).	[24]
*PCSK7* (Proprotein convertase subtilisin/kexin type 7)	rs236918 (C/G minus) G risk allele (rare) for increased levels of ferritin and soluble transferrin receptor (sTfR) and liver cirrhosis; C is the common allele	High CHO (55–65%) vs low CHO (35–45%) of low glycemic index	GG homozygotes of *PCSK7* rs236918 (rare) had a greater decrease in fasting insulin when consuming a high-CHO diet (CHO-rich foods with low glycemic index were used in the present intervention).	730 overweight or obese adults 2-year weight-loss trial	[25]
**Carbohydrates-Fibre**					
*TCF7L2* Transcription Factor 7-Like 2	rs7903146 (C/T) T risk allele for T2D; C is the common allele	High-quality CHO	Higher fiber intake may associate with protection from T2D only among *TCF7L2* rs7903146 CC allele carriers	Cohort of 24,799 non-diabetic individuals from the Malmö Diet and Cancer Study (MDCS), with dietary data obtained by a modified diet history method, follow up for 12 years, with 1,649 recordings of incident T2D made	[26]
*GCKR* Glucokinase regulatory protein	rs780094 (G/A minus) A risk allele for T2D and dyslipidemias; G is the common allele	High-quality CHO	Beneficial effects of whole-grain foods on insulin homeostasis are diminished in *GCKR* rs780094 AA risk carriers. This is possibly via the strong effect of *GCKR* variant on both triglyceride and glucose levels.	14 cohorts comprising ∼48,000 participants of European descent (meta-analysis)	[27]
**FAT**					
*PPM1K* PP2C domain-containing protein phosphatase 1K	rs1440581 (C/T minus) C risk allele. For T2D and increased BCAA /AAA ratio; T is the common allele	Low-fat diet (20% fat) vs high-fat diet (40% fat)	Individuals carrying the *PPM1K* rs1440581 C allele benefit less in weight loss and improvement of insulin sensitivity than those without this allele when undertaking an energy-restricted high-fat diet	734 overweight or obese adults 2-year weight-loss trial	[28]
*PPM1K* PP2C domain-containing protein phosphatase 1K	rs1440581 (C/T minus) C risk allele. For T2D and increased BCAA /AAA ratio; T is the common allele	Low-fat diet: 20–25% fat, 15% protein, and 60–65% carbohydrate; high-fat diet: 40–45% fat, 15% protein, and 40–45% carbohydrate	In high-fat diet, the T allele was associated with a higher reduction of insulin and HOMA-B. # The opposite effect was observed in the low-fat diet group, although in this group the T allele was marginally associated with insulin and HOMA-B,	757 nondiabetic individuals who were randomly assigned to 1 of 2 energy-restricted diets that differed in macronutrient composition	[29]
Genetic score of SNPs related to habitual coffee consumption-	8 SNP	Low-fat diet (20% fat) and high-fat diet (40% fat)	Participants genetically prone to high coffee consumption may benefit more by eating a low-fat diet in improving fasting insulin and HOMA-IR in a short term (Actual coffee consumption was not taken into account)	811 overweight or obese individuals aged 30–70 y and with a BMI (in kg/m2) of 25–40. 2-year weight-loss trial	[30]
*CLOCK* Clock circadian regulator	rs4580704 (G/T) G allele with protective effect for T2D; C is the common allele	MUFA > 13.2% of energy SFA intakes (>11.8%).	The protective effect of the *CLOCK* rs4580704 G allele on insulin sensitivity was only present when MUFA intake was >13.2% of energy. The adverse effect of C allele variant on waist circumference was only observed with high saturated fatty acid intakes (>11.8%).	Participants (*n* = 1100) in the Genetics of Lipid Lowering Drugs and Diet Network (GOLDN)	[31]
*CLOCK* Clock circadian regulator	rs1801260 (C/T) C risk allele for MetS; T is the common allele	High saturated fatty acid (SFA) intakes (>11.8%).	Individuals carrying the *CLOCK* rs1801260 risk C allele had increased waist circumference only with high saturated fatty acid intakes (>11.8%).	Participants (*n* = 1100) in the Genetics of Lipid Lowering Drugs and Diet Network (GOLDN)	[31]
Genetic risk score (GRS) SNPs related for fasting glucose-	14 SNPs	Low-fat diet (20% fat) and high-fat diet (40% fat)	Participants with a higher genetic risk may benefit more by eating a low-fat diet to improve glucose metabolism.	733 adults 2-year weight-loss trial	[32]
*CLOCK* Clock circadian regulator	rs1801260 (C/T) C risk allele for MetS; T is the common allele	(Med Diet: 35% fat, 22% monounsaturated fatty acids (MUFA)) versus low-fat diet (28% fat, 12% MUFA).	12 months of low-fat intervention, subjects who were homozygous for the common allele T displayed lower plasma insulin concentrations lower insulin resistance and higher insulin sensitivity compared with carriers of *CLOCK* rs1801260 risk allele C (TC + CC). The opposite effect observed with MedDiet although didn’t reach statistical significance	5 MetS subjects participating in the CORDIOPREV 12 month intervention	[33]
*ADIPOQ* Adiponectin ADIPOR1 Adiponectin receptor 1	*ADIPOQ* rs266729 (C/G) C risk allele for increased waist circumference; G allele protective effect against colon cancer ADIPOR1 rs10920533 (G/A) A risk allele for increased waist circumference; A is the common allele	SFA reduction	A reduction in plasma SFAs lowers insulin resistance in MetS subjects who are *ADIPOQ* rs266729 CC carriers and *ADIPOR1* rs10920533 AA carriers	451 subjects with the MetS who participated in the LIPGENE	[34]
*CLOCK* clock circadian regulator	rs4580704 (G/C) G allele with protective effect for T2D; C is the common allele	MED diet rich in MUFA	Med Diet increased the protective effects of the *CLOCK* rs4580704 G-allele against T2D and stroke	7098 PREDIMED trial (ISRCTN35739639) participants after a median 4.8-year follow-up.	[35]
*TCF7L2* Transcription Factor 7-Like 2	rs7903146 (C/T) T risk allele for T2D; C is the common allele	High dietary SFA intake (≥15.5% energy)	High dietary SFA intake (≥15.5% energy) exacerbated MetS risk and was associated with further impaired insulin sensitivity in the T allele carriers relative to the CC homozygotes and particularly to the T allele carriers with the lowest SFA intake	LIPGENE-SU.VI.MAX study of MetS cases and matched controls (*n* = 1754) Cohort of 13,000 individuals studied over 7.5 years beginning in 1994 to 2002	[36]
*LEPR* Leptin receptor	rs3790433 (G/A minus) G risk allele (common) for insulin resistance; A is rare allele	Low (plasma (*n*-3) and high (*n*-6) PUFA	Individuals with *LEPR* rs3790433 GG genotype exacerbated their risk to hyperinsulinemia and insulin resistance when their plasma levels where low for (*n*-3) and high for (*n*-6) PUFA These associations were abolished against a high (*n*-3) or low (*n*-6) PUFA background.	LIPGENE-SU.VI.MAX study of MetS cases and matched controls (*n* = 1754). Cohort of 13,000 individuals studied over 7.5 years beginning in 1994 to 2002	[37]
**PROTEIN**					
GRS related to diabetes	31 SNPs	Low-protein diet (15% protein) and high-protein diets (25% protein).	Individuals with a lower genetic risk of diabetes may benefit more from consuming a low-protein weight-loss diet in improving insulin resistance and β cell function, whereas a high-protein diet may be more beneficial for white patients with a higher genetic risk	744 overweight or obese nondiabetic adults 2-year weight-loss trial Pounds lost trial	[38]
GRS related to BMI and/or WHR	159 SNPs SNPs related to obesity abdominal obesity and T2D	Low-protein diet (15% protein) and high-protein diets (25% protein).	Participants with higher WHR only*+PGS showed less increased fasting glucose and less reduction in HOMA-B when consuming an average-protein diet, compared with lower WHR only+PGS. Conversely, eating high-protein diet was associated with less decreased HOMA-B among individuals with lower than higher WHR only+PGS *waist-hip ratio-increase only	692 overweight participants (84% white Americans) 2-year weight-loss trial Pounds lost trial	[39]
*DHCR7* 7-Dehydrocholesterol Reductase	rs12785878 (T/G) T risk allele for vitamin D deficiency G allele rare in Caucasians (no health effect)	Low-protein diet (15% protein) and high-protein diets (25% protein).	Individuals carrying the *DHCR7* rs12785878 T genotype might benefit more in improvement of insulin resistance than noncarriers by consuming high-protein weight-loss diets.	6 months (up to 656 participants) and 2 years (up to 596 participants) 6 and 2-year weight-loss trial Pounds lost trial	[40]
**MIXED DIETARY PATTERNS**					
S100 Calcium-binding protein A9 (*S100A9*)	rs3014866 (C/T) C risk allele for T2D T allele protective against diabetes.	Low SFA: CHO ratio	Individuals with the *S100A9* rs3014866 CC risk genotype may be more likely to benefit from a low SFA: carbohydrate ratio intake to improve insulin resistance as evaluated with the use of the HOMA-IR	3 diverse populations: the CORDIOPREV (Coronary Diet Intervention with Olive Oil and Cardiovascular Prevention; *n* = 711), which consisted of Spanish white adults; the GOLDN (Genetics of Lipids Lowering Drugs and Diet Network; *n* = 818), which involved North American non-Hispanic white adults; and Hispanic adults who participated in the BPRHS (Boston Puerto Rican Health Study; *n* = 1155).	[41]
*GIPR* Gastric inhibitory polypeptide receptor	rs2287019 (C/T) C risk allele for T2D T allele rare	Low-fat diet (20% fat) and high-fat diet (40% fat) High CHO (55–65%) vs low CHO (35–45%) of low glycemic index	The T allele of *GIPR* rs2287019 is associated with greater improvement of glucose homeostasis in individuals who choose a low-fat, high-carbohydrate, and high-fiber diet.	737 overweight adults 2-year weight-loss trial Pounds lost trial	[42]
*IRS1* Insulin receptor substrate 1	rs2943641 (C/T) C risk allele for T2D T allele rare	Low-fat diet (20% fat) and high-fat diet (40% fat) High CHO (55–65%) vs low CHO (35–45%) of low glycemic index	Individuals with the *IRS1* rs2943641 CC genotype obtain more benefits in weight loss and improvement of insulin resistance than those without this genotype by choosing a high-carbohydrate and low-fat diet	738 overweight adults 2-year weight-loss trial Pounds lost trial	[43]
*PLIN-1* Perilipin 1	rs894160 (G/A minus) A risk allele for increased waist circumference and T2D; G is the common allele	SFA: CHO	Women with *PLIN1* rs894160 AA genotype were more susceptible to insulin resistance in when consuming a high-saturated fat, low-carbohydrate diet. Furthermore in another study high complex carbohydrate in takes from individuals with the risk allele were protected against obesity, and the opposite was observed when consuming a low carbohydrate diet	Total of 1909 men and 2198 women (aged 18–69 years) 1 year randomized study	[44]
GRS for T2D	10 SNPs	Protein, SFA low quality food	Intakes of processed meat, red meat, and heme iron (Western dietary pattern) showed significant interactions with GRS in relation to diabetes risk. The diet-diabetes associations were more evident among men with a high GRS than in those with a low GRS.	Health Professionals Follow-Up Study (HPFS) cohort (prospective) Nested, case-control study of 1196 diabetic and 1337 nondiabetic men. 1986–2000	[45]
*TCF7L2* Transcription Factor 7-Like 2	rs7903146 (C/T) T risk allele for T2D; C is the common allele	High intake of desserts and milk	The T2D risk was greater in T allele carriers with high dessert and milk. In subjects with a high dessert intake, the T allele was also associated with higher fasting plasma glucose concentrations	787 subjects (378 men and 409women, aged between 30 and 64).	[46]

## Data Availability

Non applicable.
